# Impaired aortic distensibility and elevated central blood pressure in Turner Syndrome: a cardiovascular magnetic resonance study

**DOI:** 10.1186/s12968-018-0497-0

**Published:** 2018-12-13

**Authors:** Jan Wen, Christian Trolle, Mette H. Viuff, Steffen Ringgaard, Esben Laugesen, Ephraim J. Gutmark, Dhananjay Radhakrishnan Subramaniam, Philippe Backeljauw, Iris Gutmark-Little, Niels H. Andersen, Kristian H. Mortensen, Claus H. Gravholt

**Affiliations:** 1 Department of Endocrinology and Internal Medicine and Medical Research Laboratories, Aarhus University Hospital, Nørrebrogade 44, 8000 Aarhus C, Denmark; 2 Department of Clinical Medicine, MR Research Centre, Aarhus University Hospital, Aarhus, Denmark; 3 Department of Aerospace Engineering and Engineering Mechanics, CEAS, University of Cincinnati, Cincinnati, OH USA; 4 UC Department of Otolaryngology – Head and Neck Surgery, Cincinnati, OH USA; 5 Division of Endocrinology, Cincinnati Children’s Hospital Medical Center, University of Cincinnati College of Medicine, Cincinnati, OH USA; 6 Department of Cardiology, Aalborg University Hospital, Aalborg, Denmark; 7 Cardiovascular Imaging Department, Cardio-respiratory Unit, Great Ormond Street Hospital for Children NHS Foundation Trust, London, WC1N 3JH UK; 8 Department of Molecular Medicine, Aarhus University Hospital, Aarhus, Denmark

**Keywords:** Vascular stiffness, Hypertension, Aortic distensibility, Chromosome aberrations, Aortic dissection, Turner Syndrome

## Abstract

**Background:**

Women with Turner Syndrome have an increased risk for aortic dissection. Arterial stiffening is a risk factor for aortic dilatation and dissection. Here we investigate if arterial stiffening can be observed in Turner Syndrome patients and is an initial step in the development of aortic dilatation and subsequent dissection.

**Methods:**

Fifty-seven women with Turner Syndrome (48 years [29–66]) and thirty-six age- and sex-matched controls (49 years [26–68]) were included. Distensibility, blood pressure, carotid-femoral pulse wave velocity (PWV), the augmentation index (Aix) and central blood pressure were determined using cardiovascular magnetic resonance, a 24-h blood pressure measurement and applanation tonometry. Aortic distensibility was determined at three locations: ascending aorta, transverse aortic arch, and descending aorta.

**Results:**

Mean aortic distensibility in the descending aorta was significantly lower in Turner Syndrome compared to healthy controls (*P* = 0.02), however, this was due to a much lower distensibility among Turner Syndrome with coarctation, while Turner Syndrome without coarctation had similar distensibility as controls. Both the mean heart rate adjusted Aix (31.4% vs. 24.4%; *P* = 0.02) and central diastolic blood pressure (78.8 mmHg vs. 73.7 mmHg; *P* = 0.02) were higher in Turner Syndrome compared to controls, and these indices correlated significantly with ambulatory night-time diastolic blood pressure. The presence of aortic coarctation (*r* = **−** 0.44, *P* = 0.005) and a higher central systolic blood pressure (*r* = **−** 0.34, *P* = 0.03), age and presence of diabetes were inversely correlated with aortic distensibility in TS.

**Conclusion:**

Aortic wall function in the descending aorta is impaired in Turner Syndrome with lower distensibility among those with coarctation of the aorta, and among all Turner Syndrome higher Aix, and elevated central diastolic blood pressure when compared to sex- and age-matched controls.

**Trial registration:**

The study was registered at ClinicalTrials.gov (#NCT01678274) on September 3, 2012.

**Electronic supplementary material:**

The online version of this article (10.1186/s12968-018-0497-0) contains supplementary material, which is available to authorized users.

## Introduction

Turner Syndrome is the second most common chromosomal aneuploidy in females, occurring in 1 out of 2000 live births [1]. Women with Turner Syndrome face a 100-fold increased risk of aortic dissection [2, 3], which, along with a high incidence of coronary heart disease, hypertension, stroke and congenital heart disease may detrimentally impact life expectancy [4–6]. Factors associated with the often fatal aortic dissection include aortic dilatation [2], hypertension [7], bicuspid aortic valve (BAV) [8], 45,X karyotype, and coarctation of the aorta (CoA) [3, 9–11]. Unfortunately, these known risk factors fail to predict all events of aortic dissection [12], and there is a need to improve risk stratification beyond measuring aortic diameter and assessing aortic growth. A risk factor that considers the dynamic properties of aortic wall function could result in better prediction of dissection and rupture risk. Cardiovascular magnetic resonance imaging (CMR) can locally assess aortic distensibility. A prior study found reduced aortic distensibility in adolescents with Turner Syndrome at the level of the pulmonary artery bifurcation and the level of the diaphragm [13]. Increased arterial stiffness could therefore be a part of the pathophysiology behind the high prevalence of aortic disease in Turner Syndrome [14]. Hence, assessment of arterial stiffness may help elucidate impaired arterial function to better understand aortic disease and ultimately to improve the identification of patients with aortic dilation and hopefully to improve prognostication of this group of patients, as shown in other populations [15, 16].

The aim of this study was to investigate arterial stiffness and hemodynamic parameters, such as blood pressure, carotid-femoral pulse wave velocity (PWV), the augmentation index (Aix), central blood pressure, rate of dilation of the aorta and distensibility in the thoracic aorta in adults with Turner Syndrome, compared to healthy controls.

## Methods

### Study population

Females with karyotype-proven Turner Syndrome and age- and sex-matched controls were enrolled into this cross-sectional study from a prospective study of cardiovascular health in Turner Syndrome [11, 17, 18]. The participants had been recruited through the Danish National Society of Turner Syndrome Contact Group and an endocrine outpatient clinic. Exclusion criteria were malignancy, liver disease, contraindications to CMR, and pregnancy. Out of 67 eligible participants, fifty-seven completed this study, with exclusions due to CMR contraindications (*n* = 1), claustrophobia (n = 1), prior Bentall procedure (n = 1), suboptimal image quality with repeat CMR declined (*n* = 2), and aortic valve prosthesis (*n* = 6). Out of 39 eligible healthy age-matched controls, 36 completed the study with exclusions due to claustrophobia (*n* = 1) and suboptimal image quality with repeat CMR declined (*n* = 2). All examinations were performed during the same day.

### Pulse wave velocity (PWV) and pulse wave analysis (experiment 1)

In a first experiment pulse waves were recorded in the carotid and the femoral artery using a SphygmoCor (SPT-301B; Millar, Houston, Texas, USA) in combination with an applanation tonometer. Based on non-invasive recordings of the pulse waves, central blood pressure and carotid femoral pulse wave velocity can be determined. The investigation was performed between 7.30 A.M. and 9.30 A.M. following an overnight fast. Participants were instructed not to smoke or ingest caffeinated beverages at least 3 h before the examinations. Two of the investigators (JW and CT) performed all measurements with the patient in the supine position in a temperature-controlled room after > 5 min of rest.

#### Carotid-femoral PWV

PWV calculation is based on recording travel speed of the pulse wave generated by cardiac contraction over a known distance and is reported as meters/second. With the SphygmoCor system, the carotid PWV is calculated by so-called sequential electrocardiogram (ECG)-gated pulse wave recordings: With the patient in the supine position, three ECG electrodes were applied allowing the recording of R-waves. The distance between the suprasternal notch and the carotid pulse mark and the distance between the suprasternal notch and the femoral pulse mark were measured and entered into the SphygmoCor software. The software then subtracted the suprasternal notch-carotid distance from the suprasternal notch-femoral distance (dist_subtr). The pulse wave at the carotid artery was then recorded by the tonometer, and the time delay between the R-wave and the arrival of the pulse wave at the carotid artery was stored. Then pulse wave was recorded at the femoral artery, and the time delay between the R-wave and the arrival of the pulse wave at the femoral artery was stored. The software then subtracted the travel time of the recorded R-wave to the carotid artery from the travel time of the recorded R-wave to the femoral artery (=travel time, subtr) and calculated the carotid-femoral PWV as (dist, subtr)/(travel time, subtr). The transit time was determined by the intersecting tangent algorithm method [19], as recommended by the manufacturer (www.atcormedical.com.au/download/Active/Research_Manual_(CVMS).pdf). The SphygmoCor equipment averages the pulse wave velocity over multiple heart cycles during 10 s of recordings. Distances were measured using a slide gauge. At least two PWV measurements were obtained, accepting a standard deviation< 20% for each individual PWV measurement, which is typically composed of the average of 7–8 heart beats. Quality control excluded nine individuals with Turner Syndrome and six controls from the PWV [20], and in five individuals with Turner Syndrome and one control, we could not technically measure PWV. Fifteen individuals with Turner Syndrome and five controls were excluded from the PWV analysis due to an Operator Index ≤80, and three Turner Syndrome individuals were technically impossible to measure. Therefore, 49 participants with Turner Syndrome and 34 controls had valid data for PWV analysis.

#### Pulse wave analysis

The PWV at the radial artery was recorded non-invasively during 10 s by the Millar tonometer. Based on recordings of the radial pulse wave, blood pressure in the ascending aorta was computed using the inbuilt transfer function of the SphygmoCor software [21]. The computations were calibrated by cuff-based brachial systolic and diastolic blood pressure as recommended by the manufacturer. Blood pressure was measured by a Riester Champion N automatic blood pressure monitor three times and averaged (Riester GmbH, Jungingen, Germany). Before blood pressure measurements, arm circumference was measured with a tape measure and an appropriately sized cuff was used.

The Aix is an expression of the augmentation of the blood pressure in the ascending aorta from reflecting waves adding to the blood pressure generated from the systolic contraction. The Aix in the ascending aorta was computed based on the recordings at the radial artery. The pressure arising from the reflected waves is termed the augmentation pressure. The sum of the forward and the reflected waves is the central pulse pressure. The central Aix was calculated as the augmentation pressure divided by the central pulse pressure and thus expressed the augmentation pressure as a percentage of the pulse pressure [19, 22]. Aix was adjusted for heart rate by linear regression.

Pulse wave analysis measurements with an operator index (measure of both waveform reproducibility and signal strength) of ≤80 was excluded as recommended by the vendor [22].

### Aortic distensibility (experiment 2)

**Fig. 1:**
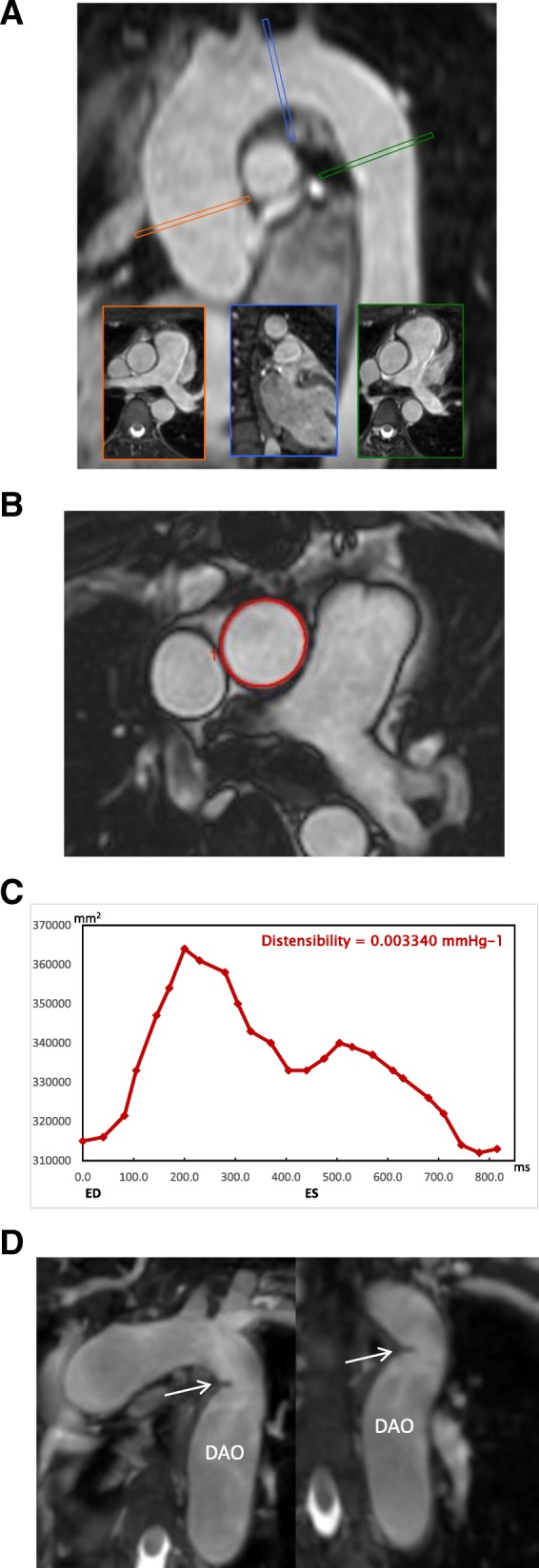
**a** Illustration of the three separate imaging slices used for balanced steady state free precession (bSSFP) cine imaging on a representative sagittal reformat of the 3D volume used for planning. The imaging slices were positioned perpendicular to the aortic wall in the mid-ascending aorta (at the level of the pulmonary bifurcation), the transverse aortic arch arch (after the left common carotid artery) and the proximal descending aorta (at the level of the pulmonary arterial bifurcation which was after any aortic coarctation in all). **b** Sample frame from a 2D bSSFP cine image for measurement of aortic distensibility in the ascending aorta, demonstrating the contouring. **c** The aortic area variation over the cardiac cycle with time (ms) on the x-axis and cross-sectional is (mm^**2**^) on the y-axis. ED – end diastole, ES – end systole. **d** The diagnosis of aortic coarctation was made from non-contrast enhanced diastolic 3D data sets used for the planning of the bSSFP cine imaging planes. The diagnosis was based on the presence of a shelf-like constriction of the aortic lumen at the aortic isthmus. This is shown here in modified sagittal and coronal reformats in a female with Turner syndrome who has luminal narrowing (arrow) with post-stenotic dilatation of the descending aorta (DAo)

At 11 A.M. CMR was performed on a 1.5 Tesla CMR (Achieva-dStream, Philips Healthcare, Best, The Netherlands) scanner using the standard anterior and posterior coils. Aortic distensibility was derived from 2D cine images acquired with a balanced steady-state-free-precession (bSSFP) sequence using retrospective electrocardiogram (ECG) gating. Repetition time was 3.6 ms and echo time was 1.8 ms. Depending on heart rate, 19 to 24 cardiac frames were acquired in a 14 s breath-hold. Slice thickness was 6 mm, field-of-view was 313 × 313 mm^2^ and pixel size was 1.6 × 1.6 mm^2^. Three imaging planes were acquired perpendicular to the aortic wall at: 1) the ascending aorta at the level of the main pulmonary artery bifurcation, 2) the transverse aortic arch, and 3) the descending aorta at the level of the main pulmonary artery bifurcation (Fig. 1a). The arterial blood pressure for distensibility calculation was measured before and after the scan with the patient lying on the scanner bed, using a CMR compatible sphygmomanometer (Aneroid, ERKA, Germany). Average blood pressure from the two measurements was used in subsequent computations. Aortic distensibility Ao*D* was calculated as:

$$ AoD=\frac{\Delta  A}{\Delta  P\bullet {A}_{dia}} $$

where *ΔA* is the difference between the maximum and minimum cross-sectional aortic area over the cardiac cycle, *ΔP* is the difference between systolic and diastolic blood pressures, and *A*_*dia*_ is the diastolic cross-sectional aortic area [23]. The cross-sectional areas were obtained by semi-automatic segmentation of the vessel through all cardiac frames using the Siswin® software (Steffen Ringgaard, Aarhus, Denmark). The vessel lumen was manually selected by a single point within the vessel in one frame, and the vessel edge was determined as the maximum image intensity gradient found radially from this point. Edge point outliers were removed, and an ellipse was fitted to the edge points. The automatic vessel selections were visually inspected and manually corrected where necessary (Fig. 1b). The variation of aortic area over the cardiac cycle is shown in Fig. 1c. All participants were examined by the same staff and in the same scanner. The scans were analysed in two bulks. Scans were analysed in duplicates following anonymization using digest package in R (3.1.0 Foundation for Statistical Computing, Vienna, Austria). One scan was analysed 10 times to determine intra-observer variability of distensibility (coefficient of variation: 10% at the ascending aorta, 8% at the aortic arch, and 11% at the descending aorta).

### Echocardiography and anatomical CMR (experiment 3)

As previously described and presented elsewhere [17, 24], transthoracic echocardiography was performed by a single observer on a GE Vivid 7 (GE Healthcare, Horten, Norway), with a 2.5 MHz transducer using second harmonic imaging. Aortic valve morphology and function were noted.

**Fig. 2:**
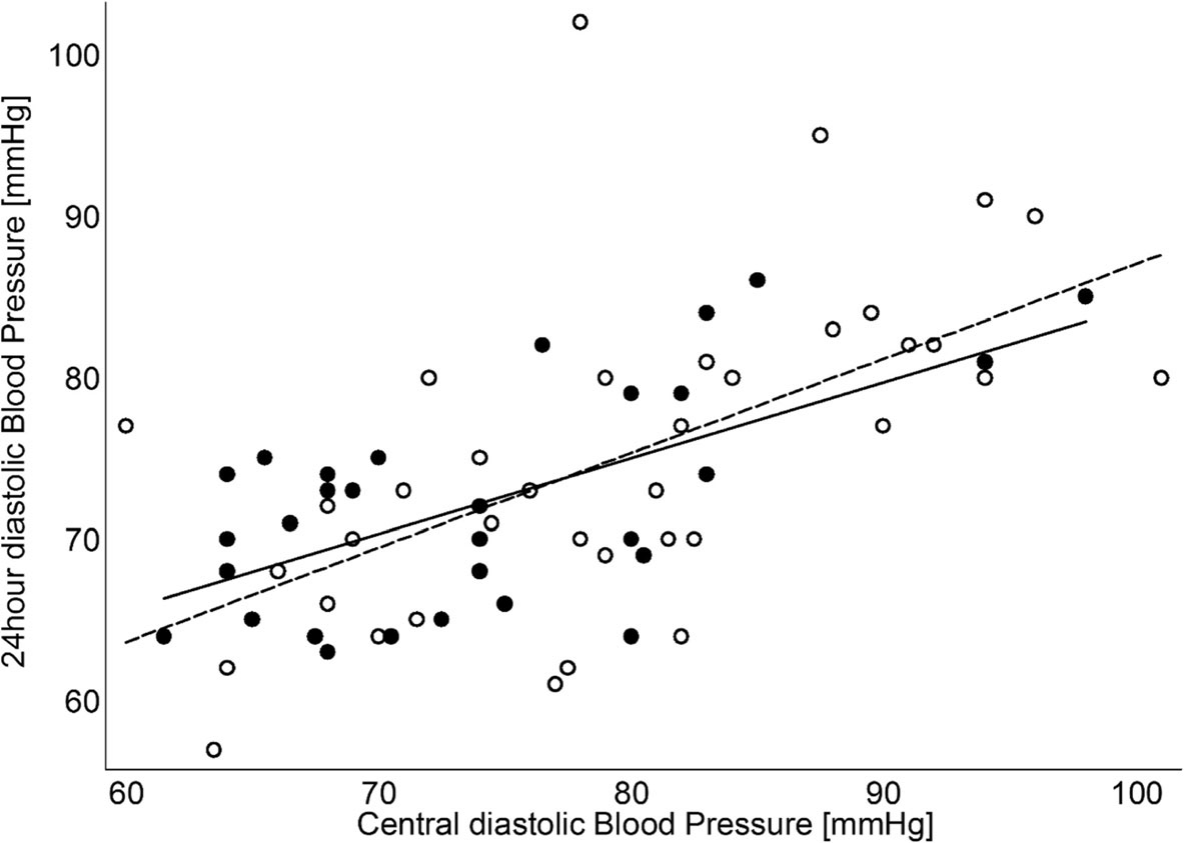
24-h diastolic blood pressure [mmHg] in women with Turner Syndrome and age and gender matched controls. Open circles and dashed linear fit are Turner Syndrome (24 h diastolic blood pressure = 28.50 + 0.59 x central diastolic blood pressure, *r* = 0.6, *P* = 0.001), and filled circles and full linear fit line are controls (24 h diastolic blood pressure = 37.47 + 0.47 x central diastolic blood pressure, r = 0.6, *P* = 0.0003)

Aortic arch anomalies were determined from a 3D bSSFP diastolic-triggered and respiratory-gated non-contrast enhanced sequence [17, 24]. Aortic arch anomalies were diagnosed as 1) aortic coarctation when there was a shelf-like narrowing of the aortic lumen in the region of the aortic isthmus (Fig. 1d), and 2) elongated transverse aortic arch when the arch appeared elongated with a kink of the inferior curvature at the aortic isthmus. Echocardiography and CMR angiography were performed to define anomalies of the aortic valve and thoracic aorta [24], which included bicuspid aortic valve (BAV) and elongated transverse aortic arch (ETA).

In addition, we have examined these Turner Syndrome women over a period of 10 years and have determined the rate of dilation of the aorta from *t* = 0 to *t* = 10 years at nine positions: (i) aortic sinuses (measuring cusp-to-opposing-cusp diameter at the point of the maximum aortic diameter in the aortic sinus); (ii) the sinotubular junction; (iii) mid-ascending aorta at the level of the inferior margin of right pulmonary artery; (iv) distal ascending aorta immediately proximal to brachiocephalic artery; (v) proximal aortic arch between the brachiocephalic and left carotid artery arteries; (vi) distal aortic arch immediately proximal to left subclavian artery; (vii) aortic isthmus immediately distal to the left subclavian artery; (viii) proximal descending aorta between the left pulmonary artery and the top of left atrium; and (ix) distal descending aorta at the most caudal border of the left atrium [18]. These data have been added to the results.

### Blood pressure and associated cardiovascular features (experiment 4)

Following experiment 2 and 3, 24 h ambulatory blood pressures were obtained with oscillometric measurements every 20 min (Spacelabs 90,217, Spacelabs Healthcare, Issaquah, Washington, USA). The cuff was placed on the left upper arm in all participants, and the cuff size was adjusted to the arm circumference. Fasting blood samples were drawn. Height, weight and medical history were recorded.

### Statistics

Statistical analysis was performed using Stata/IC 13.1 (StataCorp LP, College Station, Texas, USA). Normality was assessed by Q-Q plots of absolute or log-transformed values, and box-plots were scrutinized for outliers. Student’s independent t-test (given as mean ± SD or for transformed values, as geometric mean with confidence interval) or Mann–Whitney U-test (given as median with range) were used as appropriate. Repeated measurements were assessed using analysis of variance (ANOVA) testing the interaction between group (Turner Syndrome or control) and aortic position, reporting the Box’s conservative *P*-value. Assumptions were checked by Q-Q plot of the residuals, assessment of the covariance matrix, and assessment of sphericity. Comparisons of nominal variables were performed using the Fisher’s exact test. Bivariate correlations were assessed using Pearson’s coefficient of correlation. Explanatory models were constructed for aortic distensibility using multiple linear regression analyses. Independent variables were chosen from the bivariate correlation analyses of continuous variables. Independent variables were omitted from the models when *p* > 0.10. The contribution of each variable to the final model is stated as standardized coefficients (β). Assumptions behind the regression models were checked by Q-Q plots of the residuals, plotting residuals versus fitted, residuals versus each of the independent variables, box-plots, and leverage plots. A *p*-value < 0.05 was considered statistically significant.

## Results

The mean age for Turner Syndrome women was 47.5 years [29–66] of which 60% (*n* = 34) had 45,X monosomy and 40% (*n* = 23) were mosaics. Thirty-six healthy women, with a median age of 48.7 years [26–68; *p* = 0.9], served as controls (Table 1). Between-group differences were found for body mass index (BMI), body surface area (BSA), height, and night-time diastolic blood pressure (Table 1). Eleven women with Turner Syndrome had BAV (19%), eight (14%) had a repaired coarctation (CoA), and 26 (46%) had an ETA. Ten women with Turner Syndrome (18%) had type 2 diabetes, 28 (49%) were on antihypertensive treatment, and nine (16%) were treated with a statin. Mean plasma levels of high-density lipoproteins, low-density lipoproteins, triglycerides, and glycated haemoglobin (HbA1C) did not differ between groups. Five controls (14%) were treated for hypertension and one (3%) received a statin. None in the control group had diabetes.

**Table 1:** Anthropometrics and baseline descriptives in Turner syndrome and age and gender-matched controls, and Turner syndrome without CoA (Coarctation of Aorta) and Turner syndrome with CoA

	Turner Syndrome (*n* = 57)	Controls (*n* = 36)	*P* value†	Turner Syndrome without CoA (*n* = 49)	Turner Syndrome with CoA (*n* = 8)	*P* value‡
Age [years]	46.1 ± 10.2	46.0 ± 12.9	0.9	45.9 ± 10.2	47.1 ± 10.3	0.8
Weight [kg]	62.5 ± 14.6	71.2 ± 13.5	**0.01**	61.8 ± 14.3	66.9 ± 16.4	0.4
Height [cm]	147.2 ± 6.7	169.3 ± 6.4	**< 0.001**	147.0 ± 7.1	148.1 ± 3.8	0.7
Body Mass Index [kg/m2]	28.9 ± 6.7	24.9 ± 4.6	**0.001**	28.6 ± 6.6	30.5 ± 7.3	0.5
Body Surface Area	1.5 ± 0.2	1.8 ± 0.2	**< 0.001**	1.5 ± 0.2	1.6 ± 0.2	0.4
24 h systolic Blood Pressure [mmHg]^a^	117.9 ± 12.7	115.2 ± 10.7	0.3	117.5 ± 13.0	120.1 ± 11.6	0.6
24 h diastolic Blood Pressure [mmHg]^a^	74.5 ± 10.2	71.7 ± 6.6	0.1	74.3 ± 9.9	75.3 ± 12.5	0.8
Day-time systolic Blood Pressure [mmHg]^a^	123.4 ± 13.5	121.4 ± 10.6	0.5	122.8 ± 13.5	126.6 ± 14.2	0.5
Day-time diastolic Blood Pressure [mmHg]^a^	78.6 ± 11.4	76.9 ± 6.5	0.4	78.3 ± 10.7	80.3 ± 15.1	0.7
Night-time systolic Blood Pressure [mmHg]^a^	106.4 ± 11.9	103.3 ± 11.1	0.2	106.4 ± 12.6	106.4 ± 8.2	1
Night-time diastolic Blood Pressure [mmHg]^a^	66.2 ± 8.6	61.5 ± 7.1	**0.01**	66.3 ± 8.7	65.6 ± 8.9	0.9
Systolic Blood pressure, Sphygmocor (right)	121.3 ± 12.9	119.4 ± 13.8	0.5	120.7 ± 13.3	125.1 ± 10.0	0.4
Diastolic Blood pressure, Sphygmocor (right)	77.6 ± 9.8	72.1 ± 8.8	**0.007**	77.3 ± 10.1	79.4 ± 8.8	0.6
Systolic Blood pressure, Sphygmocor (left)	118.2 ± 13.8	118.4 ± 14.0	1.0	117.8 ± 14.3	120.6 ± 10.1	0.6
Diastolic Blood pressure, Sphygmocor (left)	76.3 ± 9.4	71.3 ± 9.0	**0.01**	76.2 ± 9.4	77.1 ± 10.3	0.8
Low Density Lipids [mmol/L]^b^	2.6 ± 0.8	2.6 ± 0.9	0.9	2.7 ± 0.8	2.6 ± 0.9	0.9
High Density Lipids [mmol/L]^b^	1.8 ± 0.6	1.8 ± 0.5	0.8	1.8 ± 0.5	1.7 ± 0.7	0.9
Triglycerides [mmol/L]^b^	1.1 ± 0.7	1.1 ± 1.0	0.8	1.1 ± 0.6	1.4 ± 0.9	0.2
HbA1C [mmol/mol]^b^	36.0 [28.0–70.0]	35.5 [26.0–48.0]	0.2	36.0 [28.0–62.0]	38.4 [31.0–70.0]	0.3
Bicuspid aortic valve^c^	19% (11/57)	0% (0/36)	**0.006**	12% (6/49)	63% (5/8)	**0.005**
Coarctation of the aorta^c^	14% (8/57)	0% (0/36)	0.02	0% (0/49)	100% (8/8)	**< 0.001**
Elongated transverse aorta^c^	46% (26/57)	0% (0/36)	**< 0.001**	43% (21/49)	63% (5/8)	0.3
Type 2 Diabetes^c^	18% (10/57)	0% (0/36)	**0.006**	12% (6/49)	50% (4/8)	**0.03**
Statins^c^	16% (9/57)	3% (1/36)	0.08	14% (7/49)	25% (2/8)	0.4
Aspirin^c^	5% (3/57)	0% (0/36)	0.3	6% (3/49)	0% (0/8)	0.6
Antihypertensives^c^	49% (28/57)	14% (5/36)	**< 0.001**	49% (24/49)	50% (4/8)	0.6
Hormone replacement treatment^c^	72% (41/57)	3% (1/36)	**< 0.001**	73% (36/49)	63% (5/8)	0.4

Blood pressure marked with SphygmoCor where used to calibrated measurements done with the SphygmoCor equipment

^a^for Turner syndrome: *n* = 50, for controls: *n* = 34. For Turner syndrome without CoA: *n* = 42

^b^for Turner syndrome: *n* = 56, for controls: *n* = 36. For Turner syndrome without CoA: *n* = 48

^c^Fisher’s Exact Test

†: *p*-value for the comparison between the entire TS cohort and controls

‡: *p*-value for the comparison between TS without CoA and TS with CoA

### Pulse wave analysis, PWV and central blood pressure (experiment 1 and 4)

Central diastolic blood pressure and night-time diastolic blood pressure were significantly raised in Turner Syndrome (Table 1), and 24-h ambulatory diastolic blood pressure correlated with central diastolic blood pressure (*r* = 0.6, *p* = 0.0001. Figure 2) and heart rate adjusted Aix. The 24-h systolic blood pressure measurements revealed comparable day and night-time values in Turner Syndrome and controls. Heart rate adjusted Aix (Table 2) was higher in Turner Syndrome compared to controls (*p* ≤ 0.03) even when excluding women with CoA, whilst PWV was comparable (*p* = 0.6).

**Table 2:** Pulse wave velocity and pulse wave analysis in Turner syndrome and healthy age-matched controls

	Turner Syndrome	Controls	*P*-value
Augmentation index [%]^b^	31.4 [27.9–34.9]	24.4 [20.3–28.5]	**0.02**
Augmentation index [%] without CoA^c^	31.3 [27.5–35.21]	24.4 [20.3–28.5]	**0.04**
Pulse wave velocity [m/s]^a^	6.76 [6.39–7.16]	6.9 [6.56–7.32]	0.6
Central systolic blood pressure [mmHg]^b^	114.3 ± 13.1	111.3 ± 16.4	0.4
Central diastolic blood pressure [mmHg]^b^	78.8 ± 9.92	73.7 ± 8.94	**0.02**

Central systolic blood pressure, central diastolic blood pressure, and Augmentation index were derived from the pulse wave analysis

^a^TS (*n* = 62), Controls (*n* = 38). ^b^TS (*n* = 49), Controls (n = 34). ^c^TS (*n* = 41), Controls (*n* = 34)

### Aortic distensibility, rate of dilation of the aorta and aortic area (experiment 2 and 3)

**Fig. 3:**
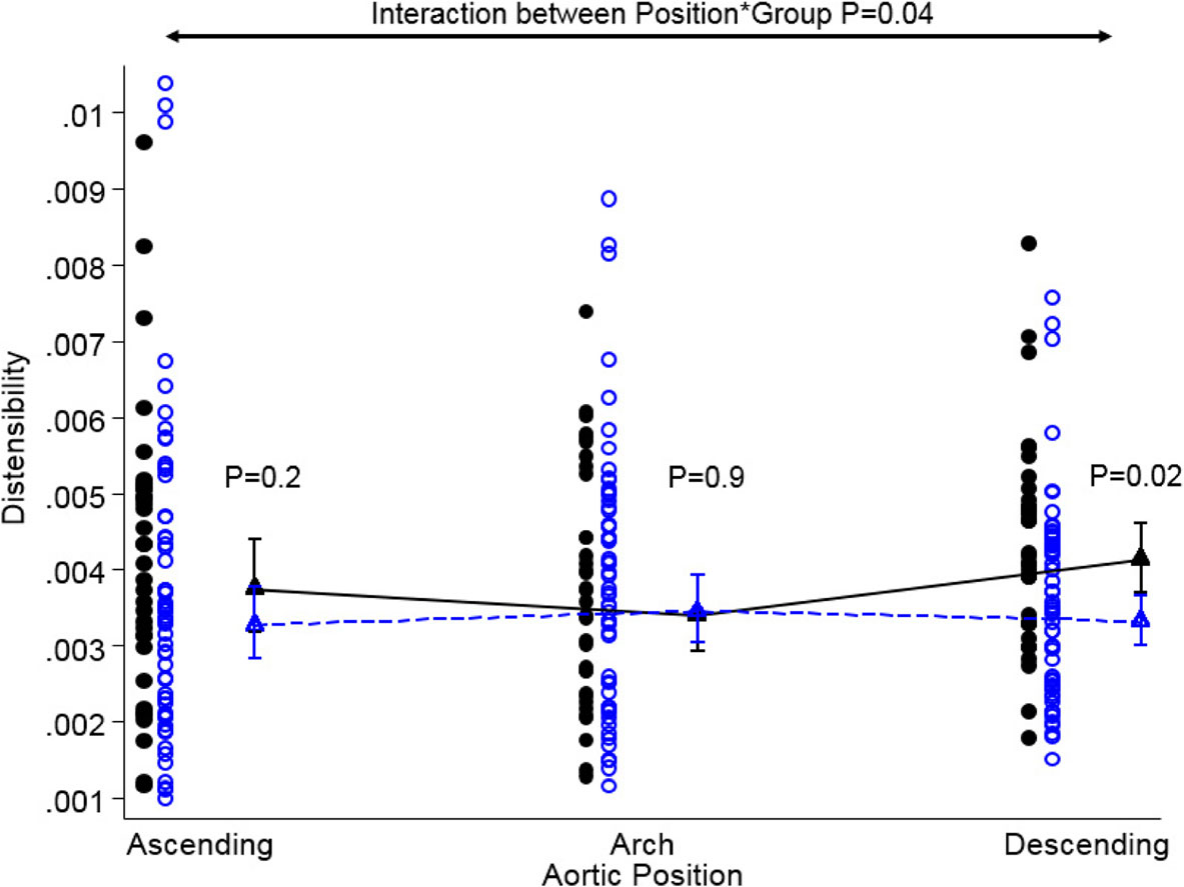
Dotplot of aortic distensibility (mmHg^− 1^) at three positions in the thoracic aorta in women with Turner Syndrome and age and gender matched controls. Triangles are geometric means with 95% confidence interval. Each dot represents an individual; blue circles are Turner Syndrome and black circles are controls. *P*-values for interaction (Group and aortic position) as well as comparison between Turner Syndrome and controls at each position are given in the plot

The overall distensibility throughout the aorta differed between Turner Syndrome and controls (interaction between group and aortic position; *p* = 0.042). There was no site-specific difference in the ascending aorta and in the aortic arch, while aortic distensibility was reduced in the descending aorta in Turner Syndrome (*p* = 0.02) (Table 3, Fig. 3). Aortic distensibility was significantly lower in women with CoA (*n* = 8), both in the ascending aorta and descending aorta when compared to those without CoA (Table 3 and Additional file 1: Figure S1). Excluding women with CoA from the comparison of Turner Syndrome and controls left only a trend towards a lower distensibility at the descending aorta (*p* = 0.1; Table 3, Additional file 1: Figure S2). Turner Syndrome subgroups divided according to karyotype (*p* = 0.5), aortic valve morphology (*p* = 0.4) and the presence of an ETA (p = 0.5) were comparable for aortic distensibility, while Turner Syndrome with type 2 diabetes had significantly lower aortic distensibility at all sites (all *p* < 0.05), also when only studying patients with CoA in the ascending aorta and arch (all *p* < 0.05).

**Table 3:** Aortic distensibility [mmHg^−1^], area (mm^2^) and area/BSA (mm^2^/(kg/m^2^)) at each of the three aortic positions in adult women with TS

	Turner Syndrome (*n* = 57)	Controls (*n* = 36)	
Distensibility			**0.042** ^a^
Ascending	3.27 [2.83–3.77]*10^−3^	3.74 [3.17–4.41]*10^− 3^	0.2
Arch	3.45 [3.04–3.92]*10^− 3^	3.39 [2.93–3.93]*10^− 3^	0.9
Descending	3.32 [3.01–3.66]*10^− 3^	4.13 [3.71–4.60]*10^− 3^	**0.02**
	TS with aortic coarctation (*n* = 8)	TS without aortic coarctation (*n* = 49)	
Distensibility			0.2^a^
Ascending	2.09 [1.34–3.26]*10^−3^	3.51 [3.03–4.07]*10^− 3^	**0.003**
Arch	2.88 [1.92–4.33]*10^− 3^	3.56 [3.10–4.08]*10^− 3^	0.2
Descending	2.25 [1.85–2.74]*10^− 3^	3.54 [3.20–3.91]*10^− 3^	**0.01**
	TS without aortic coarc. (*n* = 49)	Controls (*n* = 36)	
Distensibility			**0.05** ^a^
Ascending	3.51 [3.03–4.07]*10^−3^	3.74 [3.17–4.41]*10^− 3^	0.5
Arch	3.56 [3.10–4.08]*10^− 3^	3.39 [2.93–3.93]*10^− 3^	0.6
Descending	3.54 [3.20–3.91]*10^− 3^	4.13 [3.71–4.60]*10^− 3^	0.1
Area	TS (*n* = 60)	Control (*n* = 37)	
Ascending	758.5 [373.5–1499.9]	774.0 [505.4–1220.6]	0.3
Arch	439.3 [272.2–864.1]	497.4 [253.2–717.9]	**<0.001**
Descending	389.8 [211.2–881.2]	410.4 [226.7–711.4]	0.4
Area/BSA	TS (*n* = 60)	Control (*n* = 37)	
Ascending	490.8 [256.9–947.6]	434.5 [277.1–624.1]	0.3
Arch	279.3 [172.1–459.2]	278.4 [176.1–388.7]	0.7
Descending	252.3 [151.8–622.7]	230.4 [153.0–356.9]	0.5

First part of the table depicts comparison between all TS and controls. The two middle parts of the table depicts comparison between TS with and without aortic coarctation, and comparison between TS without coarctation and controls. *P*-value for interaction overall (group and aortic position^a^) as well as comparison between TS and controls at each position are listed. Lower part of the table depicts mean aortic area and mean aortic area corrected for BSA at the three aortic positions

### Associations between distensibility, blood pressure, rate of aortic diameter and other measures

Within the Turner Syndrome group, central systolic blood pressure (ascending: *r* = − 0.45, *p* = 0.002; descending: *r* = **−** 0.34, *p* = 0.03), CoA (*r* = − 0.30, *P* = 0.02; *r* = − 0.41, *p* = 0.0013), and Aix (*r* = − 0.35, P = 0.02; *r* = **−** 0.31, *p* = 0.04) correlated with distensibility in the ascending and descending aorta, respectively, whereas BMI, BSA and central diastolic blood pressure did not (all *p* > 0.6). Only central systolic blood pressure correlated significantly with distensibility in the aortic arch. Age was strongly correlated with aortic distensibility at all levels in both Turner Syndrome and controls (all *p* < 0.001). When including these significant variables in a multiple linear regression model of the descending aorta, only CoA and age remained significantly explanatory variables of distensibility (β = **−** 0.42, *p* = 0.001; Table 4), while only age remained a significant explanatory variable of distensibility at the level of the ascending aorta and the arch (β = **−** 0.52 and β = **−** 0.47, both *p* < 0.001).

**Table 4:** Determinants of aortic distensibility in the descending aorta in adult women with Turner syndrome

	Beta	SE	*P*-value
Central systolic blood pressure	−0.00001	0.00002	0.5
Augmentation index	−4.8^−6^	0.00002	0.8
Age	−0.0006	0.00003	**0.005**
Type 2 Diabetes	−0.0002	0.0006	0.7
Aortic coarctation	−0.001	0.0005	**0.03**

Multiple linear regression model including central systolic blood pressure, age and diabetes status, augmentation index (heart rate corrected), and aortic coarctation shows that only aortic coarctation and age remained significant explanatory variables to aortic distensibility in adult women with Turner syndrome

In separate analyses, we assessed the association between distensibility and the rate of change in aortic diameter during 10 years follow-up at anatomically-linked predefined thoracic sites. Ascending aortic distensibility did not correlate with rate of change in aortic diameter at any of the nearby assessed sites. Arch distensibility correlated with rate of change in aortic diameter at the aortic isthmus immediately distal to the left subclavian artery (*r* = − 0.36, *p* = 0.01). Finally, descending aortic distensibility correlated with rate of change in aortic diameter at proximal aortic arch between the brachiocephalic and left carotid arteries (− 0.36, *p* = 0.03) (Table 5). Omitting all cases with CoA did not materially change these results (results not shown).

**Table 5:** Correlations between site-specific aortic distensibility and rate of change of aortic diameter during the prior 10 years in adult women with Turner syndrome. *N* = 45–48

	Distensibility		
	Ascending	Arch	Descending
Aortic sinuses	*r* = 0.16, *p* = 0.3	–	–
Sinotubular junction	*r* = −0.15, *p* = 0.3	–	–
Mid-ascending aorta	*r* = 0.02, *p* = 0.9	–	–
Distal ascending aorta	*r* = 0.07, *p* = 0.7	*R* = 0.12, *p* = 0.4	–
Proximal aortic arch	–	*r* = −0.16, *p* = 0.4	***r*** **= −0.36,** ***p*** **= 0.03**
Distal aortic arch	–	r = 0.12, *p* = 0.4	*r* = 0.04, *p* = 0.8
Aortic isthmus	–	***r*** **= −0.36,** ***p*** **= 0.01**	*r* = −0.27, *p* = 0.07
Proximal descending aorta	–	–	*r* = −0.24, *p* = 0.1
Distal descending aorta	–	–	*r* = −0.17, *p* = 0.3

Mean aortic area was similar among Turner Syndrome and controls at the ascending and descending sites, while significantly smaller at the arch (Table 3). However, when adjusted for BSA, the mean areas was similar at all sites. Note, however, the much larger variation in area of the aorta at all studied sites among females with Turner Syndrome, with some having a clearly smaller area at all sites, while others had a much larger area at all sites (Table 3).

## Discussion

The principal findings of this study is a significantly reduced distensibility of the descending aorta, a higher Aix and increased central diastolic blood pressure in adult women with Turner Syndrome compared to sex- and age-matched controls. The principal determinants of the abnormal aortic wall properties in Turner Syndrome were CoA, presence of diabetes, central blood pressure, Aix and age, whereas other potential contributing factors such as a bicuspid aortic valve, BMI and BSA did not emerge as significant contributors.

Aortic distensibility is a measure of aortic elasticity, which is influenced by factors such as CoA and the grade of re-coarctation, systemic arterial hypertension and diabetes [20, 25, 26]. CoA was a principal source of significantly perturbed distensibility in the ascending and descending aorta in this cohort of adults with Turner Syndrome, which is in keeping with a previous study of CoA in individuals without Turner Syndrome that showed remodelling of the aorta with increased vascular stiffness [27, 28] and a study that showed reduced bioelastic properties of the thoracic aorta and also of left ventricular dysfunction after successful CoA repair in childhood [29]. In our study, we also found an association between aortic distensibility and presence of diabetes and age [30]. This supports the notion that CoA is part of a disease process spectrum that affects the entire thoracic aorta [31], and emphasises the need for comprehensive rather than focal aortic assessments in the diagnosis and surveillance of aortic disease in Turner Syndrome [32]. It also points towards age as a modulating variable, as well as the presence of diabetes [33]. Conclusively, when all comorbidity has been omitted, the young to middle-aged woman with Turner Syndrome may not necessarily have stiffening of the aortic wall, however the aorta of such a patient may still dilate due to pathophysiological mechanisms we are not yet aware of.

Even though aortic stiffness and carotid intima thickness possibly reflect two separate entities of vascular damage [34], the two parameters seem significantly correlated [35]. We have previously shown that women with Turner Syndrome have abnormal carotid intima thickness [36] and in this study we found reduced distensibility in Turner Syndrome women with type 2 diabetes. Therefore, one must consider whether abnormal vascular function in Turner Syndrome somehow both reflects an early atherosclerosis-type process combined with an aortopathy, associated to the presence of a CoA. On the longer term, greater arterial stiffness is associated with a higher incidence of atrial fibrillation [37]. The increased arterial stiffness will also lead to reduced impedance mismatch between aorta and the carotid arteries, spurning remodelling of the cerebral arteries and decreased ability of the cerebral circulation to adapt to changing flow needs [38]. This will increase the risk of ischemia, both acutely and chronically, as well as lacunar infarction and white matter lesions [38, 39], and thus these findings could be one among many other small steps towards the increased stroke risk seen in Turner Syndrome [6].

We have followed this cohort of Turner Syndrome females for a period of 10 years and assessed the rate of dilation during this period and included distensibility assessment at the latest examination. We found that higher distensibility in the aortic arch and in the descending aorta was related to lower rate of change at two different sites in the descending aorta, pointing towards an effect of normal distensibility in protecting the aortic wall properties, although clearly longer observation time with subsequent measurements of both distensibility, and other aortic wall properties, such as matrix remodeling and perhaps inflammation [40], as well as blood flow characteristics along the aorta, will be necessary to further examine these relations.

The reduced aortic distensibility in especially CoA affected Turner Syndrome extends the previous findings of abnormal aortic function in adolescents with Turner Syndrome [13, 41] and raises the question if impaired aortic stiffness precedes aortic dilation as in Marfan syndrome [42]. This is particularly important with the currently used risk markers failing to accurately predict cases of aortic dissection [20]. Interestingly, all other Turner Syndrome subgroups (BAV vs. tricuspid aortic valve, ETA vs. non-ETA, 45,X vs. mosaics) had comparable aortic distensibility to controls. Likewise, it is possible that the observed abnormalities of aortic distensibility are influenced by different blood flow profiles that has been demonstrated in Turner Syndrome, especially when BAV or CoA is present [43]. Contrary to the present study, a recent study of Turner Syndrome women found reduced aortic distensibility when BAV was present, which may possibly be due differences in the Turner Syndrome populations studied [44]. Here, we also show that the mean aortic area is similar or even slightly smaller (aortic arch) compared with controls, a difference that vanished after adjustment for BSA, but that the variability in aortic area is much larger, illustrated by the very wide range. This observation underscores the fact that the phenotypic spectrum of the aorta is larger among females with Turner Syndrome than among controls.

Smaller studies in children and young adults with Turner Syndrome previously showed increased PWV and a greater Aix compared to controls [45, 46]. The present study finds that adults with Turner Syndrome have a raised Aix but comparable PWV. A greater Aix indicates increased wave reflection from the periphery or earlier return of the reflected wave. Aix is inversely associated with height, and the lower height in Turner Syndrome compared to controls is an important contributor to the increased Aix observed in Turner Syndrome. Carotid-femoral PWV is an integrative measure that reflects the average arterial wall stiffness in the thoracic and abdominal aorta excluding the ascending aorta, the aortic arch and the adjacent descending aorta. Our data are in line with previous studies [45, 46] in the observation that carotid-femoral PWV is comparable between Turner Syndrome and matched controls, suggesting that this part of the aorta is not affected in Turner Syndrome. However, CMR studies evaluating this are lacking. Hypertension is common in Turner Syndrome with a particularly high prevalence in adults (up to 50%) [7, 47–49]. Consistent with this, half of the women with Turner Syndrome in our study were treated for hypertension. This resulted in comparable systolic and mean arterial pressures in Turner Syndrome and controls, possibly diminishing any difference in PWV, although the cross-sectional nature of the study and other differences between Turner Syndrome and controls, such as frequency of diabetes, precludes any firm conclusions. Others have found that PWV is not correlated with diastolic blood pressure, but with systolic blood pressure and mean arterial pressure in hypertensive individuals [50]. Medical intervention may reduce hypertensive wall stress and thus halt any age and disease related progressive perturbation in aortic wall properties [51]. However, intervention studies in Turner Syndrome are lacking [4].

The central diastolic and night-time 24-h ambulatory diastolic blood pressures were elevated in Turner Syndrome, in line with previous findings [47]. In addition to this, 24-h ambulatory diastolic blood pressure correlated well with central diastolic blood pressure. The increased diastolic pressure remains an enigma in Turner Syndrome, although it may related to the frequent left ventricular diastolic dysfunction often seen in Turner Syndrome [52]. Since increased central and peripheral diastolic blood pressure are also associated with aortic dilatation in the normal population [53], diastolic hypertension should be a target for even more aggressive treatment in Turner Syndrome, aiming at halting aortic dilatation, perhaps helping to make prophylactic aortic surgery a less frequently implemented intervention in TS.

## Limitations

The SphygmoCor device uses a generalized transfer function to derive central blood pressure from radial tonometry data. The algorithm used may not be valid in patients with abnormal arterial function such as CoA. Therefore, all calculations were done with and without the individuals with CoA, without resulting in significant change. The methodology of carotid-femoral pulse wave velocity measures the mean velocity of the pulse wave in the thoracic and abdominal aorta and iliac arteries to the femoral artery. The PWV in the aortic arch is not evaluated by this method. Hence, the carotid-femoral PWV and the aortic arch distensibility as evaluated by CMR are complementary. It would also have been advantageous to have compared PWV by SphygmoCor with PWV determined by phase contrast CMR, and in this way we could have determined regional alterations in blood flow dynamics. We plan to do this in future studies. A proportion of the study cohort, especially among females with Turner Syndrome, was being treated for conditions such as hypertension and hypercholesterolaemia. This could potentially influence the results. However, it would be unethical to investigate a similar group of people without treatment, and the fact that these women were receiving their usual treatment makes this study a reflection of a real-life outpatient clinic cohort.

## Conclusion

Adult Turner Syndrome females have impaired aortic wall function of the ascending and descending aorta, with reduced aortic distensibility among those with CoA, and higher central and peripheral night-time diastolic blood pressures as well, along with an elevated Aix. The principal determinant of the abnormal aortic wall function was the presence of type 2 diabetes, CoA and age. Measurement of aortic distensibility is a promising tool in future clinical care of Turner Syndrome in the prediction of cardiovascular events.

## Additional file

Additional file 1: **Table S1.** Aortic distensibility in women with Turner Syndrome but no aortic coarctation compared to healthy age and gender matched controls. Overall model *P*-value < 0.001. No significant interaction between group and position (*P* = 0.08) and hence the same development in distensibility through the aorta. Only a trend towards a lower distensibility at the descending aorta. **Figure S1.** Aortic distensibility according to the presence of aortic coarctation in Turner Syndrome. Dotplot of aortic distensibility (mmHg^− 1^) at each of the three aortic positions. Each dot represents an individual; blue circles are Turner Syndrome with aortic coarctation and black filled are indicate Turner Syndrome without aortic coarctation. Triangles are geometric means with 95% confidence interval. **Figure S2.** Aortic distensibility in women with Turner Syndrome but no aortic coarctation compared to healthy age and gender matched controls. Dotplot of aortic distensibility (mmHg^− 1^) at each of the three aortic positions. Each dot represents an individual; blue circles are Turner Syndrome without aortic coarctation and black filled are indicate controls Triangles are geometric means with 95% confidence interval. (DOCX 97 kb)

## Abbreviations

Aix: Augmentation index
AoD: Aortic distensibility
BAV: Bicuspid aortic valve
BMI: Body mass index
BSA: Body surface area
bSSFP: Balanced steady state free precession
CMR: Cardiovascular magnetic resonance
CoA: Coarctation of the aorta
ECG: Electrocardiogram
ETA: Elongated transverse aortic arch
PWV: Pulse wave velocity
