# Supplementary material for: Impaired aortic distensibility and elevated central blood pressure in Turner Syndrome: a cardiovascular magnetic resonance study
Source: J Cardiovasc Magn Reson. 2018 Dec 13;20:80. doi: 10.1186/s12968-018-0497-0 (PMC6292015; doi:10.1186/s12968-018-0497-0)
Supplement: Supplementary file 1 — Table S1. Aortic distensibility in women with Turner Syndrome but no aortic coarctation compared to healthy age and gender matched controls. Overall model P-value < 0.001. No significant interaction between group and position (P = 0.08) and hence the same development in distensibility through the aorta. Only a trend towards a lower distensibility at the descending aorta. Figure S1. Aortic distensibility according to the presence of aortic coarctation in Turner Syndrome. Dotplot of aortic distensibility (mmHg− 1) at each of the three aortic positions. Each dot represents an individual; blue circles are Turner Syndrome with aortic coarctation and black filled are indicate Turner Syndrome without aortic coarctation. Triangles are geometric means with 95% confidence interval. Figure S2. Aortic distensibility in women with Turner Syndrome but no aortic coarctation compared to healthy age and gender matched controls. Dotplot of aortic distensibility (mmHg− 1) at each of the three aortic positions. Each dot represents an individual; blue circles are Turner Syndrome without aortic coarctation and black filled are indicate controls Triangles are geometric means with 95% confidence interval. (DOCX 97 kb) [file 12968_2018_497_MOESM1_ESM.docx]

# Additional file 1

|  | Turner syndrome without aortic coarctation (n=49) | | | Controls (n=36) | | | P-value |
| --- | --- | --- | --- | --- | --- | --- | --- |
| Distensibility |  |  |  |  |  |  |  |
| Ascending | 3.51*10^-3^ |  | (3.03*10^-3^-4.07*10^-3^) | 3.74*10^-3^ |  | (3.17*10^-3^-4.41*10^-3^) | 0.5 |
| Arch | 3.56*10^-3^ |  | (3.10*10^-3^-4.08*10^-3^) | 3.39*10^-3^ |  | (2.93*10^-3^-3.93*10^-3^) | 0.6 |
| Descending | 3.54*10^-3^ |  | (3.20*10^-3^-3.91*10^-3^) | 4.13*10^-3^ |  | (3.71*10^-3^-4.60*10^-3^) | 0.1 |

Table S1. Aortic distensibility in women with Turner syndrome but no aortic coarctation compared to healthy age and gender matched controls. Overall model P-value <0.0001. No significant interaction between group and position (P=0.08) and hence the same development in distensibility through the aorta. Only a trend towards a lower distensibility at the descending aorta.

**Figure S1.** **Aortic distensibility according to the presence of aortic coarctation in Turner syndrome.** Dotplot of aortic distensibility (mmHg^-1^) at each of the three aortic positions. Each dot represents an individual; blue circles are Turner syndrome with aortic coarctation and black filled are indicate Turner syndrome without aortic coarctation. Triangles are geometric means with 95% confidence interval.

Figure S2. Aortic distensibility in women with Turner syndrome but no aortic coarctation compared to healthy age and gender matched controls. Dotplot of aortic distensibility (mmHg^-1^) at each of the three aortic positions. Each dot represents an individual; blue circles are Turner syndrome without aortic coarctation and black filled are indicate controls Triangles are geometric means with 95% confidence interval.
